# Rapid Treatment of Acute Decompensated Heart Failure with Cardiogenic Shock in the Emergency Setting

**DOI:** 10.26502/fccm.92920505

**Published:** 2026-09-07

**Authors:** Fihr Chaudhary, Sahil Afzal, Nasr Chaudhary, Devendra K Agrawal

**Affiliations:** 1 Department of Translational Research, College of Osteopathic Medicine of the Pacific, Western University of Health Sciences, Pomona, California 91766 USA; 2 University of California, Merced, CA 95343, USA; 3 The San Joaquin Valley PRIME+ BS-MD Program, University of California, Merced, CA 95343, USA

**Keywords:** Acute decompensated heart failure, Cardiogenic shock, Emergency department, Hemodynamic phenotyping, Mechanical circulatory support, Non-invasive ventilation, Norepinephrine, Point-of-care ultrasound, SCAI SHOCK classification, Shock team

## Abstract

Acute decompensated heart failure accounts for more than a million United States hospitalizations each year, and its most severe expression, cardiogenic shock, carries an in-hospital mortality approaching fifty percent. Most randomized evidence in heart failure concerns the chronic outpatient population, which leaves the emergency department phase, during which a large share of the eventual trajectory is determined, supported by comparatively few dedicated trials. This review draws together what is currently known about the recognition and early stabilization of acute decompensated heart failure complicated by cardiogenic shock in the emergency setting. It describes the shock spiral, in which falling cardiac output lowers coronary perfusion pressure and further impairs contractility, together with the inflammatory component that explains why a subset of patients present with an inappropriately low systemic vascular resistance. It reviews bedside phenotyping along the axes of perfusion and congestion, severity staging with the SCAI SHOCK classification, and the diagnostic tools available at the bedside, including point-of-care ultrasound of the lungs, heart, and inferior vena cava, the electrocardiogram as a means of identifying the precipitating arrhythmia, and the laboratory markers of perfusion and end-organ stress. The management section argues that sequence is itself a therapeutic decision: perfusion pressure is restored first, contractility second, and congestion third, because each intervention is actively harmful when performed out of order. Non-invasive ventilation, rate and rhythm management, and correction of anemia are addressed as parallel contributors to oxygen delivery. The review closes with criteria for escalation to mechanical circulatory support and multidisciplinary shock team care, and states plainly the unequal quality of the evidence supporting each recommendation.

## Introduction

An increasing number of people are being hospitalized due to acute decompensated heart failure (ADHF), which is linked to high rates of mortality and morbidity. Predictions made by the American Heart Association indicate that the prevalence of heart failure would exceed eight million cases by 2030, with total direct expenses linked to the condition increasing from $21 billion in 2012 to $70 billion in 2030 [1]. The sudden appearance or alteration of heart failure symptoms and signs is known as acute decompensated heart failure. It typically results in hospitalization and demands urgent medical treatment because it can be a life-threatening condition. Both the incidence and severity of acute decompensated heart failure are on the rise, and the disease is a leading cause of death and disability. Every year, more than one million individuals in the United States end up in the hospital with heart failure as their main diagnosis. Another three million patients have heart failure as a secondary or tertiary diagnosis [2–6]. Hospitalization for adults over the age of 65 is most caused by heart failure, and around sixty days after discharge the readmission rate reaches 35% [2–8]. Acute heart failure (HF) can manifest in various forms, the most severe of which is cardiogenic shock (CS). This condition is marked by a drastically decreased cardiac output, insufficient end-organ perfusion, and subsequent tissue hypoxia. Mortality and morbidity rates for patients with CS are extremely high [9], with in-hospital mortality reaching up to 50% [10]. While the exact mechanisms of the pathogenesis of CS are still unclear, inflammation is widely believed to play a significant role [11]. The management of acute decompensated heart failure has only lately attracted the attention of researchers, despite the abundance of large-scale randomized controlled trials involving patients with chronic HF. The consequence is a body of evidence in which the emergency department phase of care rests on comparatively few dedicated trials, even though this is the phase during which a large share of the eventual trajectory is determined. The purpose of this review is to draw together what is currently known about the recognition and early stabilization of ADHF complicated by cardiogenic shock in the emergency setting. The discussion covers the pathophysiology of the shock spiral, bedside phenotyping and severity classification, the diagnostic tools available at the bedside, the sequencing of respiratory and hemodynamic support, the management of the precipitants that most often tip a compensated patient into shock, and the criteria for escalating to mechanical circulatory support and multidisciplinary shock team care.

## Pathophysiology: The Shock Spiral

**Figure 1:**
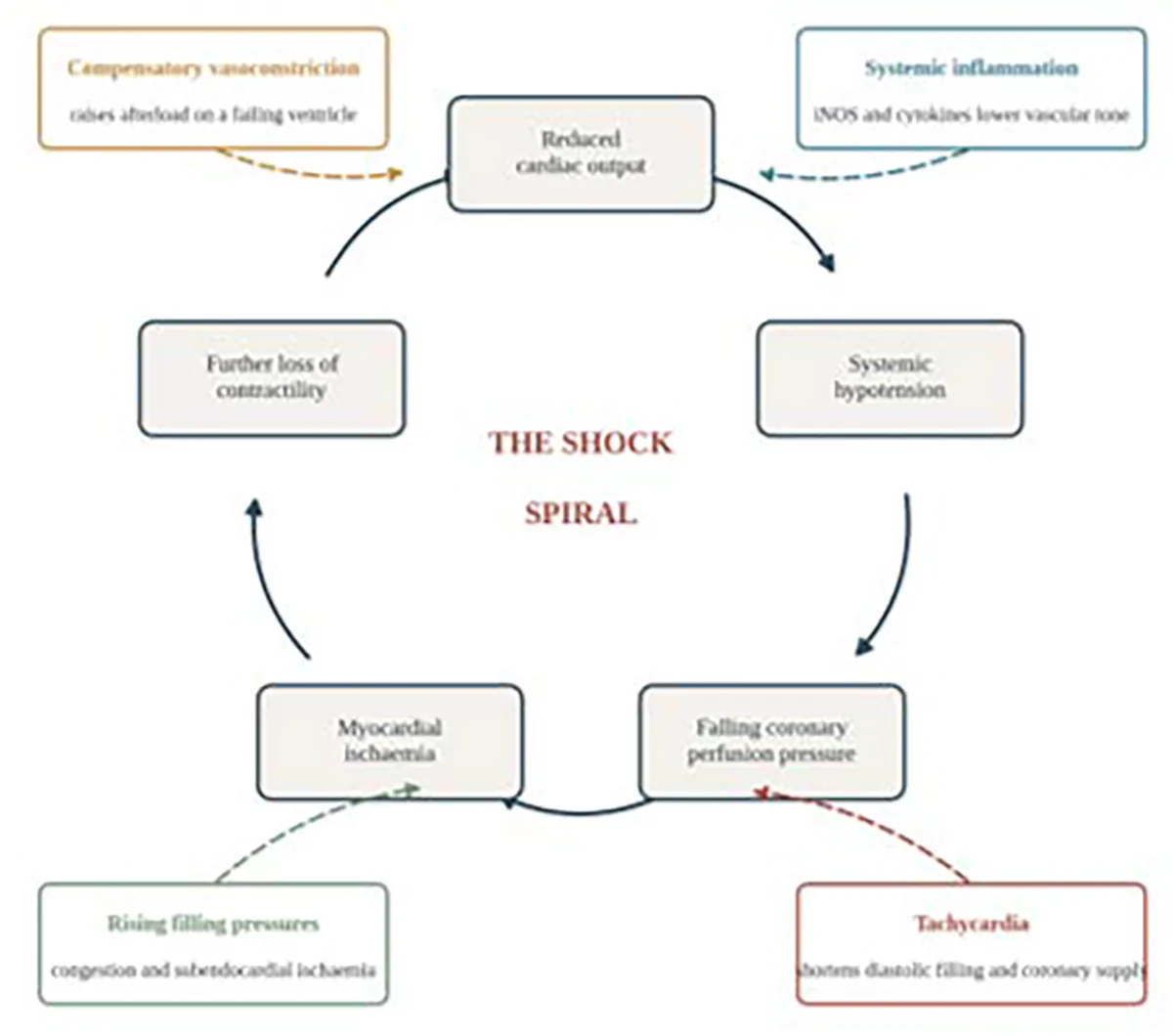
The cardiogenic shock spiral. Falling cardiac output lowers systemic pressure, which lowers coronary perfusion pressure, which worsens myocardial ischemia and further reduces contractility, closing the loop. Four external processes shown in the outer boxes feed additional injury into the cycle rather than forming part of it.

The heart enters what has been described as a death spiral when low cardiac output (CO) leads to systemic hypotension, which reduces coronary perfusion, further weakening the myocardium (Figure 1). Compensatory systemic vasoconstriction raises afterload against a ventricle that is already failing, and the resulting rise in filling pressures worsens both pulmonary congestion and subendocardial ischemia. Superimposed on this mechanical problem is a systemic inflammatory component. Increased inducible nitric oxide synthase activity and circulating cytokines produce inappropriate vasodilation in a subset of patients, which helps explain why some patients in cardiogenic shock present with a low rather than an elevated systemic vascular resistance [11,12].

Low cardiac output is recognized clinically well before it is measured. Clammy, cold skin is characteristic of low cardiac output, since the cutaneous circulation is shut down to shunt blood elsewhere, and a narrow pulse pressure follows from a stroke volume too small to generate a strong pressure wave. A weak pulse in a hypotensive patient therefore carries more information than the numerical blood pressure alone. Because the tissues of a heart failure patient are not receiving enough oxygenated blood, they transition from aerobic to anaerobic metabolism, and a lactate level greater than 2.0 mmol/L is the biochemical finding most often treated as the smoking gun indicating low CO. A creatinine that rises acutely above a known baseline indicates that cardiac output has become inadequate to sustain glomerular filtration. Hypotension in this setting is not simply a number to be corrected; it is the mechanism through which the spiral propagates. Recent studies have suggested that the hypotension experienced during vasovagal syncope may be due to a decrease in cardiac output rather than vasodilation [13]. During shock, arrhythmias, or severe vasodilation, hypotension can occur and results in a decrease in coronary perfusion pressure [14]. A drop in diastolic blood pressure, brought on by hypotension, reduces perfusion pressure and, in turn, myocardial oxygen supply [15]. In obstructive coronary artery disease (CAD), diastolic hypotension worsens coronary perfusion pressure, leading to myocardial ischemia and subclinical myocardial damage [16]. Multiple cohorts have validated the link between hypotension and an elevated risk of cardiac damage, including diabetics [17] and those with [18] and without preexisting cardiovascular disease [19]. Taken together, this body of work explains why restoring perfusion pressure, rather than relieving congestion, is the first hemodynamic priority in the emergency department.

## Clinical Phenotyping: Hemodynamic Profiles at the Bedside

ADHF can be categorized into hemodynamic stages depending on cardiac index (CI) and pulmonary capillary wedge pressure. The scheme originates with the four hemodynamic subsets described in acute myocardial infarction, which were defined by a cardiac index above or below 2.2 L/min/m2 and a pulmonary capillary pressure above or below 18 mm Hg [20]. The same two axes were subsequently translated into a purely bedside assessment that requires no invasive monitoring [21]. Cardiac index stands for degree of perfusion [22]; patients are classified as warm or cold based on the existence of hypoperfusion. A cardiac index below 2.2 L/min/m2 is considered cold, suggesting hypoperfusion and the inability of the heart to fulfill the body’s metabolic demands [23]. Symptoms that are indicative of hypoperfusion include a lack of energy, low blood pressure, cold extremities, impaired kidney function, and mental decline [24]. In cases when edema is present or absent, patients are categorized as wet or dry accordingly [25]. Volume overload manifests itself clinically with several symptoms, including breathing difficulties, dyspnea during sleep, peripheral edema, ascites, hepatomegaly, and splenomegaly, as well as a raised jugular venous pressure [26].

**Figure 2:**
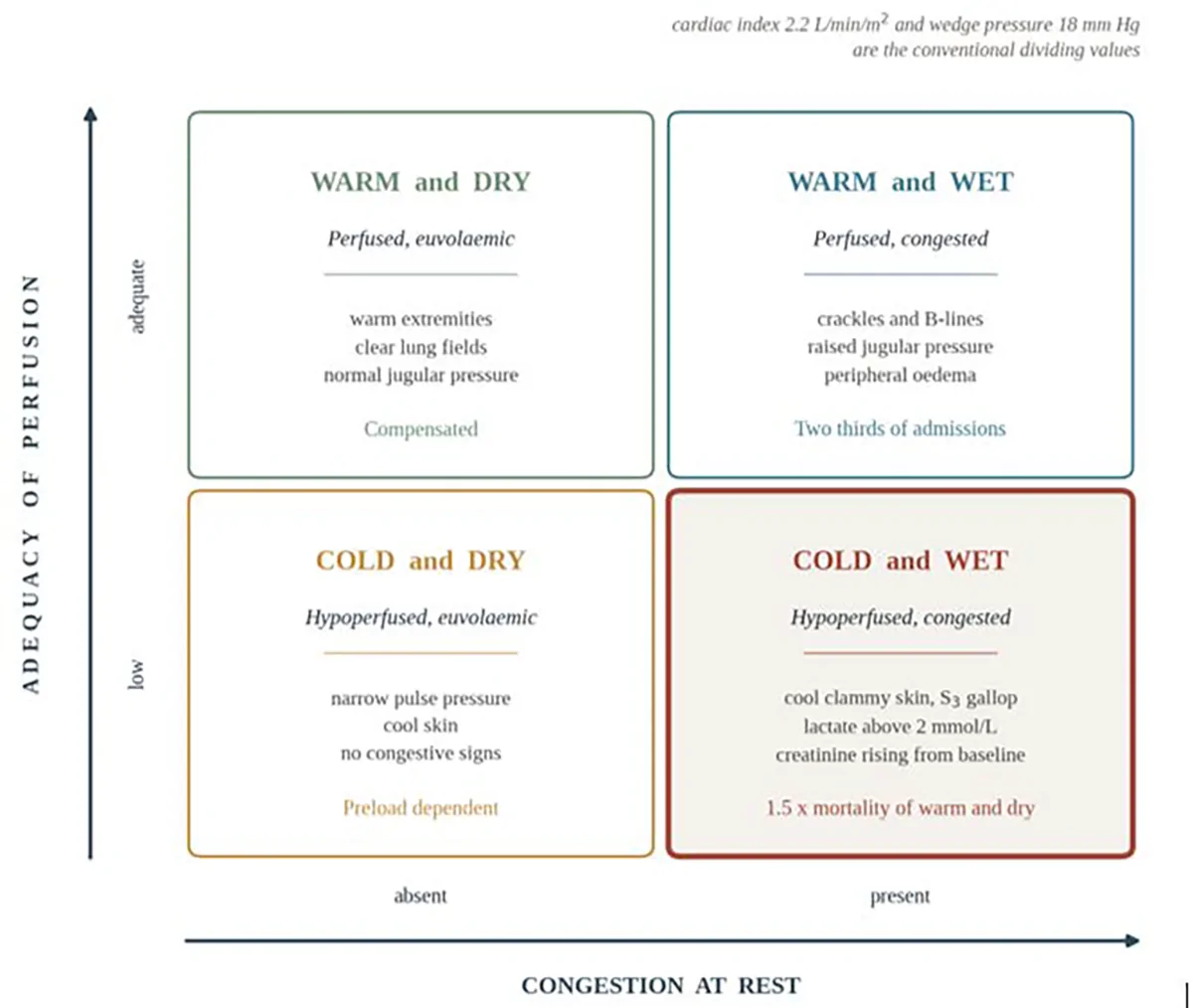
Bedside classification of acute decompensated heart failure by adequacy of perfusion and presence of congestion. Each quadrant lists the findings that assign a patient to it, together with its epidemiological or prognostic significance. The cold and wet quadrant, highlighted, is the profile in which decongestion cannot safely precede restoration of perfusion pressure.

The distribution of these profiles matters for triage. A wet and warm presentation is the most prevalent, accounting for two-thirds of ADHF hospitalizations [27]. The subgroup that is cold and wet has a mortality rate that is 1.5 times higher than warm and dry [28]. The prognostic value of the classification was established in a study of patients admitted with advanced heart failure, in which profiles assigned at the bedside predicted subsequent outcomes [28]. The four resulting profiles, together with the bedside findings that define them, are summarized in Figure 2. Successive updates to the European Society of Cardiology heart failure guidelines, in 2016 and again in 2021, have both emphasized the use of this classification in acute heart failure to direct early therapy and provide prognostic data [29, 30]. Current North American guidance likewise recommends that patients presenting with acute heart failure be triaged according to the adequacy of perfusion and the presence of congestion before therapy is selected [31].

The practical value of the phenotype is that it dictates the order of operations. A warm and wet patient can be decongested directly. A cold and wet patient cannot, because diuresis in the setting of inadequate forward flow will reduce preload without improving perfusion and will frequently precipitate worsening renal function. Recognizing the cold and wet phenotype in the first minutes of the encounter is therefore the single decision that most strongly shapes everything that follows.

## Severity Classification: SCAI SHOCK Stages

Phenotyping identifies the physiology; staging quantifies the severity. The Society for Cardiovascular Angiography and Interventions (SCAI) SHOCK classification classify patients into five stages, from a patient at risk of shock through to refractory shock. Hypoperfusion was typically defined as stage C in most studies when lactate levels were elevated to 2 mmol/L or above. Poor renal function was frequently cited as evidence of hypoperfusion, but there was a lack of research that differentiated between acute and chronic renal impairment [32]. A patient in SCAI stage C has hypoperfusion that requires intervention beyond volume, which in practice means inotropes, vasopressors, or mechanical support. Validation of the SCAI shock stages was carried out using data from 10,004 consecutive cardiac intensive care unit patients at the Mayo Clinic. The results showed that, after accounting for known mortality predictors, the likelihood of in-hospital mortality increased with each higher stage [33]. The importance of evaluating perfusion in a group of patients with severe heart failure had been detailed earlier in the study of bedside hemodynamic profiles [28]. The staging system is useful in the emergency department for two reasons that are separable from its prognostic value. It provides a common vocabulary for the handoff between emergency medicine, cardiology, and critical care, and it establishes a threshold at which escalation should be considered rather than deferred.

## Bedside Diagnostics: The Emergency Department Workup

### Point-of-Care Ultrasound (POCUS).

An accessible, user-friendly, and dependable tool for a precise ADHF diagnosis is point-of-care ultrasound. When diagnosing heart failure, POCUS has been shown to be more accurate than chest X-ray and clinical examination alone [34]. Its advantage is greatest in exactly the patients in whom the physical examination is least reliable, including those with severe obesity, chronic lung disease, or an inability to sit forward. A structured protocol that interrogates the lungs, the heart, and the inferior vena cava in sequence has been shown to yield a rapid and accurate diagnosis in undifferentiated acute respiratory failure [35].

### Lungs.

Lung ultrasound is conventionally performed over eight regions of the thorax. The presence of interstitial fluid is confirmed by diffuse, bilateral B-lines, also known as comet-tail artifacts. These findings are often linked to diseases such as pulmonary edema or pneumonia [36], and in a patient with a wet clinical presentation and bibasilar crackles they substantially raise the probability of cardiogenic pulmonary edema. Comet-tails, or B-lines, are hyperechoic reflections that arise from a single point and travel in a direction almost perpendicular to the pleural line. They move in tandem with respiration, have a thin base, and project a beam of light down the screen from the transducer [37]. When a patient breathes, the visceral and parietal pleura rub against each other, a phenomenon known as lung sliding, which on ultrasound looks like tiny ants marching on a line. Absence of lung sliding raises the possibility of pneumothorax, although the sign is not specific and is lost in a range of other conditions encountered in patients with acute respiratory failure [38]. In the dyspneic hypotensive patient, its main value is that it takes seconds to check and excludes a cause of obstructive shock that would otherwise be managed very differently.

### Cardiac Function.

Left ventricular ejection fraction (LVEF) can be estimated visually or measured from bedside M-mode acquisitions taken from the parasternal short axis using the Teichholz method. When the LVEF is significantly lower than normal, the heart is not pumping blood as efficiently as it should and underlying heart failure is likely. The usual range for LVEF is 50% to 75%, and a value below 40% is consistent with heart failure [39]. Chamber dilatation, the size and collapsibility of the inferior vena cava, and a qualitative assessment of right ventricular size and function should be obtained in the same study, since isolated right ventricular failure produces a shock state that responds poorly to the strategy appropriate for left-sided failure.

### Electrocardiography.

The electrocardiogram in a patient with decompensation serves two purposes. It screens for acute ischemia, and it identifies the arrhythmia that frequently serves as the precipitant. Atrial flutter with 2:1 conduction, which produces a ventricular rate near 150 beats per minute, is a classic example and is easily mistaken for sinus tachycardia when the flutter waves are buried in the T waves. The sawtooth waveform of atrial flutter can usually be seen in the inferior leads II, III, and aVF if one looks closely, and the rapid atrial rate can also be appreciated in V1. The clinical significance of a tachyarrhythmia in this setting is that the loss of atrial contribution to ventricular filling, combined with the shortened diastolic filling time imposed by the rate, can cause cardiac output to fall sharply. In a patient with preexisting ventricular dysfunction, such a tachyarrhythmia is often the tipping point into cardiogenic shock.

## Laboratory Analysis: Markers of Perfusion and End-Organ Stress

### Lactate.

Lactate levels reveal underlying tissue hypoperfusion and anaerobic metabolism [40]. A value above 2 mmol/L is the threshold used in most shock classification schemes, and the trajectory over the first hours of resuscitation carries more information than any single value.

### Natriuretic peptides.

An elevated B-type natriuretic peptide should prompt consideration of acute strain on the ventricles [41]. The diagnostic utility of a bedside assay in the undifferentiated dyspneic emergency department patient was established in a prospective study of 1,586 patients, in which natriuretic peptide measurement outperformed clinical judgment alone in distinguishing heart failure from other causes of breathlessness [42]. The interpretation of an absolute value requires context, since obesity lowers natriuretic peptide levels and renal impairment raises them; comparison against a patient’s own prior values, when available, is more informative than comparison against a population cutoff.

### Troponin.

A troponin within the normal range argues against acute myocardial infarction as the primary driver of decompensation, although mild elevations are common in cardiogenic shock of any cause and reflect demand ischemia rather than plaque rupture.

### Renal function.

A creatinine that rises acutely from a known baseline indicates type 1 cardiorenal syndrome, a condition in which acute kidney injury occurs because of inadequate cardiac output [43]. The five-part classification of cardiorenal syndromes distinguishes this acute, heart-driven pattern from the chronic and kidney-driven types, and the distinction matters at the bedside because type 1 physiology improves with restoration of forward flow rather than with volume [44]. Interpretation of a single creatinine in the acute setting is limited by the kinetics of the marker itself, which lags the injury by hours [43,44].

### Hemoglobin.

Anemia in a patient with heart failure is not an incidental finding. Oxygen delivery is the product of cardiac output and arterial oxygen content, so patients with both a low cardiac index and a low hemoglobin have two multiplicative deficits rather than one. Anemia and iron deficiency are common comorbidities in heart failure and are independently associated with worse clinical status and worse outcomes [45]. A patient whose pump has been supported but whose blood carries half the oxygen it should has not been fully resuscitated.

## Stepwise Emergency Management

**Figure 3:**
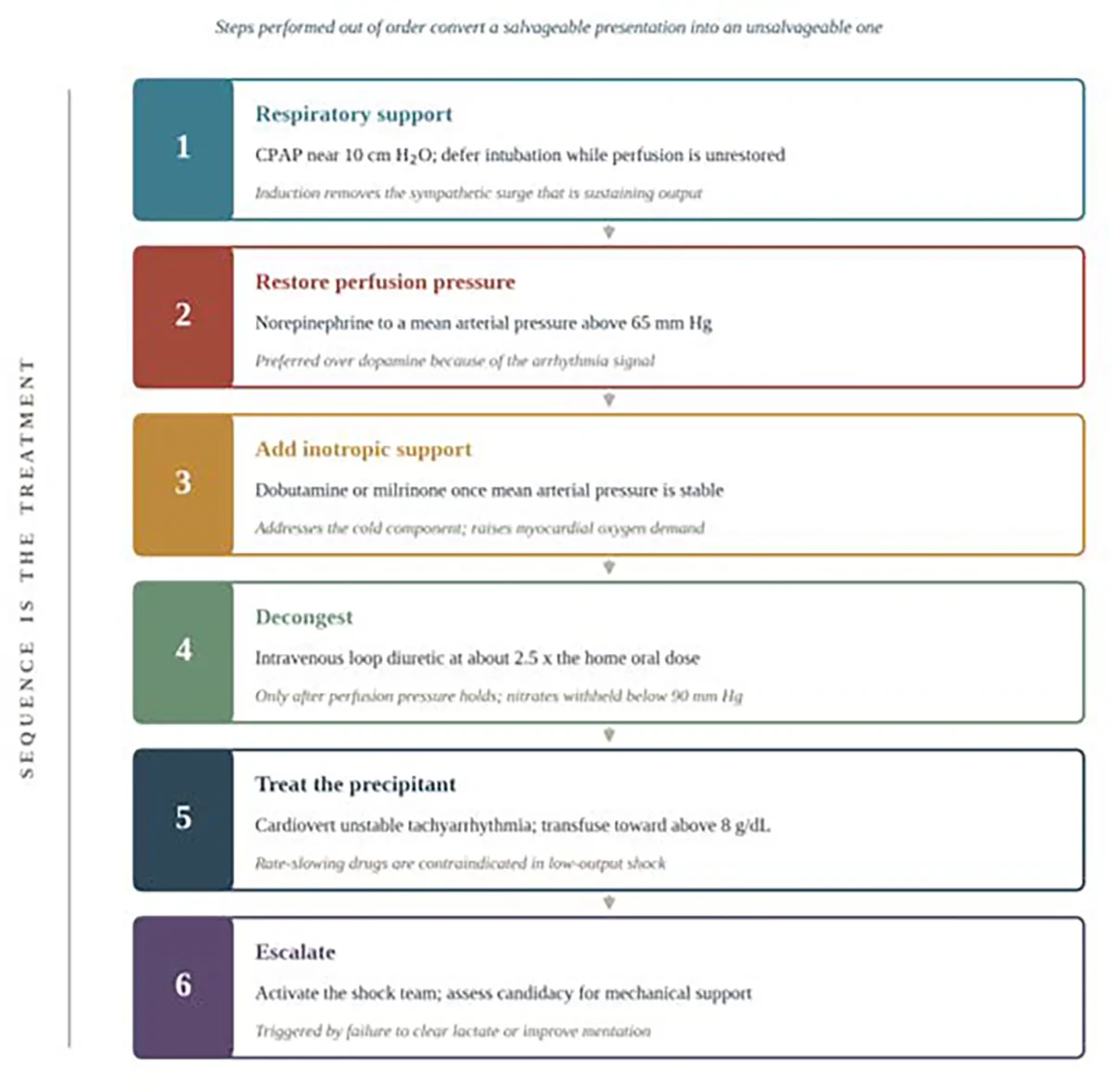
Ordered sequence of emergency department interventions in acute decompensated heart failure complicated by cardiogenic shock. The italicized line beneath each step states the constraint that determines its position in the sequence. The ordering is derived from the physiology and from current guideline recommendations rather than from a randomized comparison of sequences.

Managing the hypotensive, congested, tachycardic patient requires walking a fine line. Supporting blood pressure while simultaneously attempting to reduce heart rate and increase oxygen delivery is a challenging task, and the interventions that address one of these goals frequently worsen another. The sequence in which they are undertaken therefore matters as much as the choice of agent (Figure 3).

### Respiratory Support:

Balancing Oxygenation and Hemodynamics. Acute hypoxia and tachypnea require rapid intervention. Continuous positive airway pressure (CPAP), typically initiated around 10 cm H2O, raises intrathoracic pressure, which reduces both preload and afterload while recruiting alveoli to improve oxygenation. Research indicates that the rate of intubation can be decreased by implementing early CPAP in the emergency department [46]. A prospective study comparing out-of-hospital CPAP with standard care in patients experiencing acute respiratory distress found that it lowered the intubation rate and improved mortality [47]. Compared with traditional oxygen treatment, the rapid improvement of acute respiratory failure and metabolic disturbance is seen when positive airway pressure is applied early in patients with acute cardiogenic pulmonary edema, and endotracheal intubation becomes less necessary as a result [48]. A Cochrane review of non-invasive positive pressure ventilation in cardiogenic pulmonary oedema likewise found reductions in the need for intubation, with a probable reduction in hospital mortality and no excess of adverse events [49].

Intubation strategy deserves separate consideration in cardiogenic shock. Switching to positive pressure ventilation and administering induction medications removes the sympathetic surge that is sustaining perfusion, which not infrequently leads to rapid cardiovascular collapse. The strategy of using non-invasive ventilation as a bridge, and deferring intubation until perfusion has been restored, avoids the most dangerous peri-intubation period wherever the patient’s mental status and work of breathing permit it.

### Hemodynamic Optimization.

The first step in ensuring that all organs, and particularly the already stressed kidneys and brain, receive adequate blood flow is to stabilize the mean arterial pressure (MAP). Norepinephrine is generally the first agent used for the purpose of maintaining a MAP above 65 mm Hg. In distributive shock it is the first-line agent for maintaining mean arterial pressure throughout resuscitation [50], and gradual dose escalation has been shown to improve tissue oxygenation and cutaneous microvascular flow [51]. In individuals with cardiac systolic dysfunction, norepinephrine had neutral or positive effects on hemodynamics without adverse consequences [52]. Its comparative advantage over dopamine is well established: in a randomized trial of 1,679 patients with shock, 28-day mortality did not differ between the agents, but arrhythmic events were substantially more frequent with dopamine, and in the prespecified subgroup with cardiogenic shock dopamine was associated with higher mortality [53]. This is a decisive consideration in a patient whose shock was precipitated by a tachyarrhythmia in the first place.

Once the MAP has been stabilized, an inotropic infusion is added to address the cold component of the shock state by raising contractility and stroke volume. Dobutamine increases blood pressure, particularly the systolic reading, and heart rate [54]. Although it has only modest chronotropic effects at low to medium doses, it considerably increases myocardial oxygen demand, which is the reason it is used as a pharmacological stress agent for diagnostic perfusion imaging [55]. Its effects in shock states have been reviewed in detail and remain incompletely characterized by randomized evidence [56]. The choice between dobutamine and milrinone has been directly tested: in a double-blind randomized trial of 192 patients with cardiogenic shock, the two agents produced no significant difference in the composite primary outcome or in in-hospital mortality [57]. In the emergency department the practical distinctions are pharmacokinetic rather than outcome-based, since milrinone has a longer half-life and is renally cleared, which makes it harder to titrate in a patient with evolving acute kidney injury.

Historically, dopamine was the standard treatment for low output states. Nevertheless, norepinephrine has been found to be associated with a substantially reduced risk of arrhythmias [56]. Results from one trial showed that compared with the norepinephrine group, the dopamine group had a substantially greater mortality rate [58], whereas a later study found no statistically significant differences in either short-term or long-term death rates between the norepinephrine and dopamine groups [59]. The weight of the evidence, and particularly the arrhythmia signal, favors norepinephrine.

### Decongestion.

Even in a patient who is unambiguously wet, aggressive diuresis must wait until the MAP has been stabilized. Loop diuretics reduce preload in a patient whose cardiac output is already preload-dependent and giving them before perfusion pressure is restored converts a congested patient into a congested and underfilled one. Once perfusion has been restored, intravenous loop diuretic dosing at roughly 2.5 times the patient’s home oral dose achieves greater symptomatic relief than a low-dose strategy, at the cost of a transient rise in creatinine that does not appear to carry a durable renal penalty; bolus and continuous infusion strategies performed equivalently [60]. Nitroglycerin, although effective at reducing afterload in ADHF, is contraindicated at the low systolic pressures that define this population [31].

### Addressing the Triggers:

#### Arrhythmia and Anemia.

A common and underappreciated pattern is the double hit of tachycardia and severe anemia. Atrial fibrillation and flutter are the most prevalent sustained arrhythmias in adults and frequently occur together in the same patient [61]. A sustained rate near 150 beats per minute is metabolically expensive, because myocardial oxygen demand rises while diastolic filling time, and therefore coronary supply, falls. Atrial fibrillation decreases cardiac power and myocardial blood flow and is associated with higher myocardial oxygen extraction, meaning the heart must work harder to obtain oxygen from the blood delivered to it [62].

Rate control in this population is constrained. In a patient with poor ejection fraction and frank hypoperfusion, beta-blockers and non-dihydropyridine calcium channel blockers are contraindicated, since their negative inotropy will worsen an output problem that the tachycardia is only partly responsible for. It is worth noting that the meta-analytic evidence supporting beta-blockade in chronic heart failure does not extend to patients in atrial fibrillation, in whom no prognostic benefit was demonstrated [63]; there is accordingly no reason to accept the acute hemodynamic cost. Where the arrhythmia is judged to be the primary driver of shock, synchronized electrical cardioversion is the intervention that addresses the problem without a negative inotropic penalty, and current guidance supports its use in hemodynamically unstable patients [31].

Anemia is corrected for the same reason the pump is supported, namely, to raise oxygen delivery. Transfusion of packed red blood cells targeting a hemoglobin above 8 g/dL is a reasonable approach in a patient with hemodynamic instability and cardiovascular disease and represents a departure from the restrictive threshold of 7 g/dL appropriate for stable hospitalized adults [64]. Correction of anemia in heart failure is not a definitive therapy, and erythropoiesis-stimulating agents have not improved outcomes in this population [45], but in the acute setting the objective is narrower: restoring oxygen-carrying capacity in a patient whose delivery is already compromised by a low cardiac index.

## Escalation: Shock Teams and Mechanical Circulatory Support

When the pharmacological bridge fails to clear lactate or improve mentation, the hemodynamic ladder must escalate to mechanical circulatory support (MCS). One meta-analysis found that using a microaxial pump to unload the left ventricle before increasing the dosage of vasopressors decreased myocardial oxygen demand, because the pump continuously removes blood from the left ventricle, reducing both the workload on the ventricle and the oxygen demand of the myocardium [65]. The same device improves mean arterial pressure without increasing the requirement for catecholamines, which limits their adverse effect on the microvasculature, because it delivers substantial volumes of blood to the aorta [65]. When these actions are combined, they improve systemic perfusion and coronary flow and ultimately improve perfusion to important organs [65]. Early research using this device demonstrated that myocardial perfusion and cardiac workload were both improved by continuously emptying the left ventricle throughout the cardiac cycle [66]. Observational outcome data have been encouraging. A total survival rate of 74% to explant was achieved in patients with cardiogenic shock in the first 200 American cases utilizing one microaxial device [67]. In a subsequent single-center series, 81% of patients survived to discharge following implantation, and no patient who underwent a heart transplant using the device as a bridge died during the first ninety days following release [68]. Most of these patients had already received other forms of mechanical circulatory support and were supported to progress to advanced surgical heart failure treatments, to assist recovery or decision-making, or to prepare for high-risk cardiac surgery [68]. Randomized evidence has been more equivocal and deserves to be read alongside the registry data. In infarct-related cardiogenic shock, routine use of a microaxial flow pump reduced all-cause mortality at 180 days compared with standard care, at the cost of a higher incidence of adverse events including bleeding and limb ischemia [69]. By contrast, routine early venoarterial extracorporeal life support in the same population did not reduce 30-day mortality and increased complications [70]. The practical implication for the emergency physician is that mechanical support is a decision about patient selection and timing rather than a reflex, and that the decision belongs to a team.

Evidence suggests that shock teams composed of emergency medicine, cardiology, and critical care reduce mortality. Many high-mortality illnesses, including trauma, cardiac arrest, sepsis, and stroke, are better treated with team-based approaches [71]. The first team-based approach to cardiogenic shock was detailed at the Mayo Clinic in Arizona, where a team including a cardiac surgeon, perfusionists, and critical care nurses traveled into the community to treat patients with refractory circulatory collapse using venoarterial extracorporeal membrane oxygenation before transferring them to a tertiary center; patients stabilized in this way had a 56% chance of survival to discharge compared with 30% among those stabilized at the community hospital alone [72]. A 30-day mortality reduction from 53% to 23% was linked to the implementation of a telephone-activated multidisciplinary shock team and an institutional strategy for cardiogenic shock care at the Inova Heart and Vascular Institute in Virginia [73]. At the University of Ottawa, researchers found that a smartphone-activated shock team improved long-term survival, with 8-month mortality falling from 67% to 43% [74]. These single-center findings have since been supported by a multicenter registry analysis in which patients with cardiogenic shock managed in cardiac intensive care units with shock teams had lower mortality than those managed in units without them [75].

## Discussion

The literature reviewed here converges on a small number of practical propositions. The first is that cardiogenic shock is rarely a pure pump problem. Rhythm, oxygen-carrying capacity, and ventricular function each contribute to oxygen delivery, and a patient may have deficits in all three simultaneously. Supporting the pump while ignoring a hemoglobin of 7 g/dL or a ventricular rate of 150 beats per minute treats one term of a product and leaves the others untouched.

The second is that sequence is a therapeutic decision. Perfusion pressure is restored first, contractility second, and congestion third, because the interventions appropriate to each step are actively harmful when performed out of order. Diuresis before perfusion pressure has been restored, or afterload reduction in a patient with a systolic pressure below 90 mm Hg, converts a salvageable presentation into an unsalvageable one. This ordering is not itself the subject of a randomized trial, which is a limitation of the evidence base worth stating plainly, but it follows from the physiology and is reflected in current guideline recommendations [30,31]. Third, the evidence for the individual components of early management is uneven in quality. The comparison between norepinephrine and dopamine rests on a large, randomized trial with a clear arrhythmia signal [53], and the comparison between dobutamine and milrinone rests on a smaller trial that found no difference [57]. Non-invasive ventilation has consistent randomized support for reducing intubation [49]. Mechanical circulatory support has one positive randomized trial in a narrowly selected infarct-related population [69] and one negative trial of a different modality in the same population [64], alongside a much larger body of observational data that is subject to selection bias. Shock teams have consistent observational support and no randomized evidence [75]. These are not equivalent grades of certainty and should not be treated as such.

Fourth, several questions remain open. It is not established which patients benefit from early mechanical support as opposed to a longer trial of pharmacological therapy, nor whether the mortality benefit observed in infarct-related shock extends to shock arising from decompensated chronic heart failure, which is a physiologically distinct population that has been underrepresented in trials. The optimal MAP target in cardiogenic shock has not been determined, and the value of 65 mm Hg is extrapolated largely from the septic shock literature. Finally, the contribution of each individual element of a shock team protocol, as distinct from the protocol, has not been isolated.

A limitation of this review should be acknowledged. Much of the highest-quality evidence in cardiogenic shock is drawn from infarct-related shock, and its applicability to patients presenting with acutely decompensated chronic heart failure is an assumption rather than a demonstrated fact. Registry data suggest these populations differ in age, comorbidity burden, and trajectory, and the reader should weight the recommendations accordingly.

## Conclusion

Cardiogenic shock complicating acute decompensated heart failure is rarely attributable to a single mechanism. The recognition of the cold and wet phenotype, the assignment of a severity stage, and the identification of the precipitant are three separate cognitive tasks, and all three can be completed at the bedside within the first minutes of an emergency department encounter. What follows from them is a sequence: perfusion pressure before contractility, contractility before decongestion, and early involvement of the team that will make the decision about mechanical support. The interventions available in the emergency department are not merely supportive but prognostic, and the physician who performs them well changes the trajectory of the intensive care unit stay that follows.

## Key Points

- Cardiogenic shock complicating acute decompensated heart failure is rarely a pure pump problem, since rhythm, oxygen-carrying capacity, and ventricular function each contribute to oxygen delivery and frequently fail together in the same patient.
- The shock spiral is self-propagating: low cardiac output lowers systemic and therefore coronary perfusion pressure, which worsens ischemia and contractility, so restoring perfusion pressure rather than relieving congestion is the first hemodynamic priority.
- Bedside phenotyping along the two axes of perfusion and congestion assigns a patient to one of four profiles within minutes of arrival and is the single decision that most strongly shapes everything that follows.
- A cold and wet patient cannot be decongested first, because diuresis in the setting of inadequate forward flow reduces preload without improving perfusion and frequently precipitates worsening renal function.
- The SCAI SHOCK stages quantify severity, predict in-hospital mortality in stepwise fashion, and give emergency medicine, cardiology, and critical care a shared vocabulary and a defined threshold at which escalation should be considered rather than deferred.
- Point-of-care ultrasound is more accurate than chest radiography and clinical examination alone for the diagnosis of heart failure, and its advantage is greatest in precisely the patients whose physical examination is least reliable.
- A lactate above 2 mmol/L, a creatinine rising acutely from a known baseline, natriuretic peptides interpreted against body habitus and renal function, and hemoglobin together define a perfusion and end-organ picture that the monitor does not display.
- Norepinephrine is preferred to dopamine for restoring mean arterial pressure given a clear arrhythmia signal against dopamine and higher mortality in the cardiogenic shock subgroup, while dobutamine and milrinone performed equivalently in a randomized comparison, making that choice pharmacokinetic rather than outcome based.
- Early non-invasive positive pressure ventilation reduces the need for intubation and probably hospital mortality, and deferring intubation until perfusion has been restored avoids the peri-intubation collapse that follows removal of the sympathetic surge.
- Escalation to mechanical circulatory support is a decision about patient selection and timing rather than a reflex, and the observational literature consistently favors managing these patients through a multidisciplinary shock team.
